# Diagnostics for filovirus detection: impact of recent outbreaks on the diagnostic landscape

**DOI:** 10.1136/bmjgh-2018-001112

**Published:** 2019-02-07

**Authors:** Devy M Emperador, Laura T Mazzola, Betsy Wonderly Trainor, Arlene Chua, Cassandra Kelly-Cirino

**Affiliations:** 1 FIND, Emerging Threats Programme, Geneva, Switzerland; 2 FIND, Geneva, Switzerland; 3 Médecins Sans Frontières (MSF), Geneva, Switzerland

**Keywords:** ebolavirus, marburgvirus, in vitro diagnostics, outbreak

## Abstract

Ebolaviruses and Marburg virus (MARV) both belong to the family *Filoviridae* and cause severe haemorrhagic fever in humans. Due to high mortality rates and potential for spread from rural to urban regions, they are listed on the WHO R&D blueprint of high-priority pathogens. Recent ebolavirus outbreaks in Western and Central Africa have highlighted the importance of diagnostic testing in epidemic preparedness for these pathogens and led to the rapid development of a number of commercially available benchtop and point-of-care nucleic acid amplification tests as well as serological assays and rapid diagnostic tests. Despite these advancements, challenges still remain. While products approved under emergency use licenses during outbreak periods may continue to be used post-outbreak, a lack of clarity and incentive surrounding the regulatory approval pathway during non-outbreak periods has deterred many manufacturers from seeking full approvals. Waning of funding and poor access to samples after the 2014–2016 outbreak also contributed to cessation of development once the outbreak was declared over. There is a need for tests with improved sensitivity and specificity, and assays that can use alternative sample types could reduce the need for invasive procedures and expensive equipment, making testing in field conditions more feasible. For MARV, availability of diagnostic tests is still limited, restricted to a single ELISA test and assay panels designed to differentiate between multiple pathogens. It may be helpful to extend the target product profile for ebolavirus diagnostics to include MARV, as the viruses have many overlapping characteristics.

**Summary box**

- Ebolaviruses and Marburg virus (MARV), both of the family *Filoviridae*, cause severe haemorrhagic fever in humans and are considered high-priority pathogens by WHO; recent ebolavirus outbreaks in Western and Central Africa have led to rapid development of a number of diagnostic tests for ebolaviruses.
- Despite these advancements, gaps in diagnostic preparedness for ebolaviruses remain, including difficulties obtaining full regulatory approval for tests approved under emergency licenses, lack of incentive to continue development after an outbreak is over, poor specificity and sensitivity of some existing tests, and a need for tests able to use different sample types.
- For MARV, availability of diagnostic tests is still limited; target product profiles for ebolavirus diagnostics should be extended to include MARV and should consider persisting challenges to diagnostic preparedness for ebolaviruses.

## Introduction

**Figure 1:**
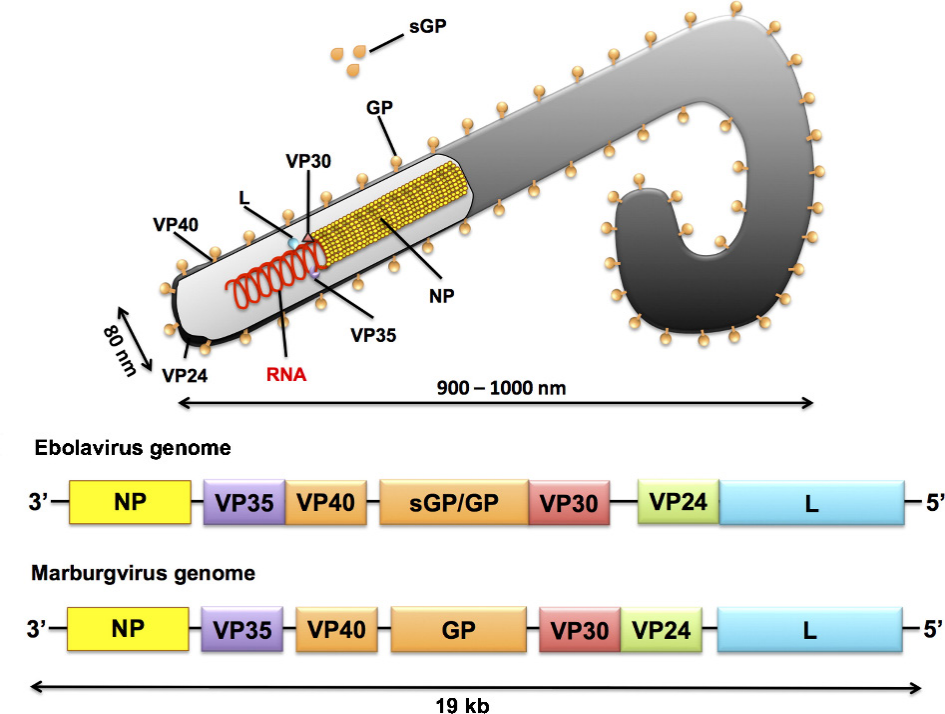
Schematic illustration of filovirus particle and ebolavirus and MARV genomes. *Reprinted from J Clin Virol, 64, Rougeron V et al., Ebola and Marburg haemorrhagic fever, 111–9. Copyright (2015), with permission from Elsevier.* GP, glycoprotein; L, non-structural protein RNA-dependent RNA polymerase; MARV, Marburg virus; NP, nucleoprotein, sGP, secreted glycoprotein; VP24, minor matrix protein; VP30, replication-transcription protein; VP35, polymerase cofactor; VP40, matrix protein.

Ebolaviruses and Marburg virus (MARV) belong to the viral family *Filoviridae* and can cause severe haemorrhagic fever in humans and non-human primates. Filoviruses are filamentous, negative-sense RNA viruses, with a genome that encodes seven structural proteins: nucleoprotein (NP), polymerase cofactor (VP35), matrix protein (VP40), glycoprotein (GP), replication-transcription protein (VP30), minor matrix protein (VP24) and the non-structural protein RNA-dependent RNA polymerase (L) (figure 1).^1^ The virus family *Filoviridae* is divided into three genera, Ebolavirus, Marburgvirus and Cuevavirus. Within the ebolavirus genus are five species: Zaire ebolavirus (EBOV), Sudan ebolavirus (SUDV), Bundibugyo ebolavirus, Tai Forest ebolavirus (TAFV) and Reston ebolavirus. The marburgvirus genus consists of Marburg marburgvirus (MARV), including the MARV POPP, MARV Angola, MARV Durba, MARV Ozolin and MARV Musoke variants, and Ravn virus.^2 3^

**Figure 2:**
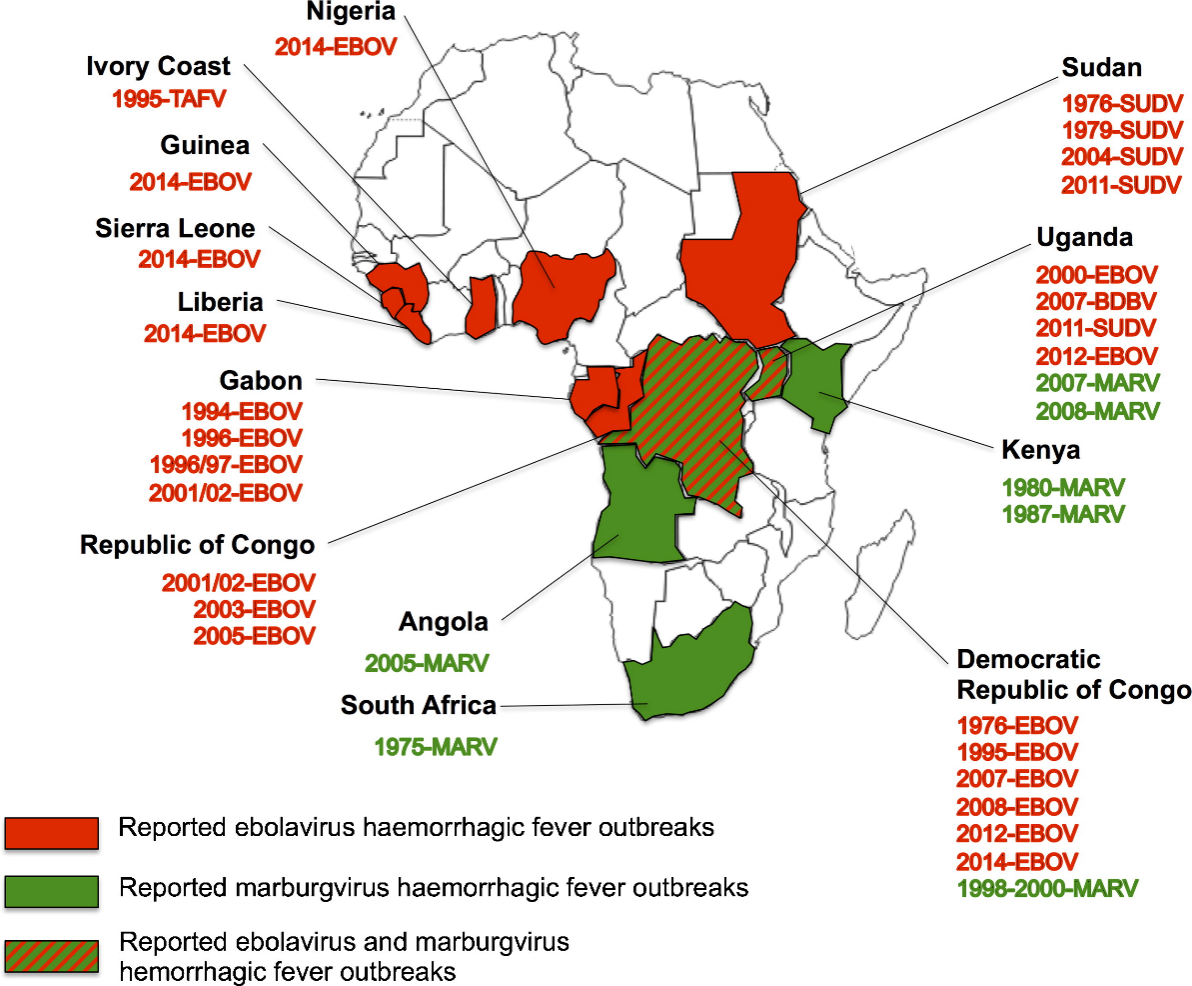
Reported outbreaks or isolated cases of MARV and ebolaviruses. *Reprinted from J Clin Virol, 64, Rougeron V et al., Ebola and Marburg haemorrhagic fever, 111–9. Copyright (2015), with permission from Elsevier.* EBOV, Zaire ebolavirus; MARV, Marburg virus; SUDV, Sudan ebolavirus; TAFV, Tai Forest ebolavirus.

Since the first report in Sudan and Zaire in 1976, ebolavirus disease (EVD) has caused more than 30 outbreaks in the subsequent 40 years (figure 2). EVD is a lethal illness with an average case fatality rate of 78%. Marburg virus disease (MVD) was first identified in 1967 during epidemics in Marburg and Frankfurt in Germany and Belgrade in the former Yugoslavia from the importation of infected monkeys from Uganda.^4^ Similar to EVD, MVD has a very high case fatality rate, measuring just over 80% in some of the most recent outbreaks.

Sporadic outbreaks of EVD and MVD typically occur and are limited to countries in sub-Saharan Africa.^4^ However, in 2014, an outbreak of EBOV was detected in rural Guinea, near the border of Liberia and Sierra Leone, resulting in 28 616 total cases of EVD and 11 310 deaths in 10 countries, with a mortality rate between 30% and 70% in Guinea, Liberia and Sierra Leone by 2016.^5^ The most recent outbreak is located in the Democratic Republic of Congo, with 137 total cases, 106 confirmed cases and 92 deaths (61 confirmed) reported as of 12 September 2018.^6^

The fruit bat is thought to be the native host for filoviruses (although this has not been definitively demonstrated for ebolaviruses),^7^ with a large ecological reach ranging from West and Central Africa to Southeast Asia.^8–13^ Reports show a seasonality in filovirus transmission to humans that may correspond with mating and birthing seasons of fruit bat species.^14 15^ Primates and other animals can also be infected and suffer disease, although their ability to serve as a reservoir for filoviruses is unknown.^9^

Filoviruses are primarily transmitted to humans through close contact with blood, secretions, organs, or other bodily fluids of infected humans or animals.^8 15–17^ They are commonly spread among family and friends of infected individuals, although nosocomial transmission, especially among healthcare workers, occurs.^18^ As seen in the 2014–2016 ebolavirus outbreak, an increase in population size, the rise of urbanisation and the interconnectedness of travel can expand the spread of filovirus disease beyond endemic regions.^19^ Isolation of patients, proper use of personal protective equipment (PPE) and disinfection procedures have been effective in reducing human-to-human transmission of EBOV and MARV.^16^

The incubation period for EVD and MVD is between 2 and 21 days, with an average of 3–10 days.^8^ Both ebolaviruses and MARV can be clinically detected in blood after onset of fever, which accompanies the rise in circulating virus within the patient’s body and remains elevated in individuals who progress to death, especially among those with EVD.^20^

Treatment options for EVD and MVD are limited and rely on supportive therapy.^8 21^ While there are no proven effective drug treatments for EVD or MVD, experimental therapies (ZMapp, brincidofovir, TKM-Ebola and favipiravir) were used during the 2014–2016 ebolavirus outbreak to treat infected patients.^21 22^ A number of EVD pharmacotherapies and immunological-based agents have been or are currently undergoing accelerated human trials in EVD endemic countries, including favipiravir and ZMapp; some may also be effective for MARV (although ZMapp is specific to ebolavirus).^21–23^

Two of the most promising EBOV vaccine candidates, rVSV-EBOV and ChAd3-EBO-Z, underwent phase II/III efficacy trials in Liberia, Sierra Leone and Guinea during the peak of the 2014–2016 epidemic, showing high efficacy and long-term antibody responses.^24–26^ Vaccines for MARV have not seen a similar accelerated development to EBOV, although animal and early clinical studies show potential for MARV vaccine immunogenicity and protection.^2 27 28^

Due to the sporadic nature of outbreaks, high mortality rates and potential spread from rural to urban regions, filoviruses are some of the high-priority pathogens identified in the WHO R&D Blueprint,^29^ a global strategy and preparedness plan to strengthen the emergency response to highly infectious diseases. To catalyse diagnostic development for filoviruses, the Foundation for Innovative New Diagnostics (FIND, www.finddx.org) has launched initiatives for needs assessment and partnerships across a broad range of diseases in endemic-prone countries. This landscape analysis describes the current state of filovirus diagnostics, and identifies remaining needs for further research and development.

## Filovirus diagnostics

The diagnosis of ebolaviruses and MARV is based on direct identification of the viral particles, proteins or specific RNA in a suspected case from whole blood (WB), serum or plasma. Since ebolaviruses and MARV are classified as biosafety level 4 agents due to their propensity for human-to-human transmission, high risk for laboratory-acquired infections, and lack of a specific and safe vaccine, patient samples present an extreme biohazard risk and special laboratory procedures must be in place to safely confirm infection.

Confirmation can only be obtained by virus isolation. However, other assays such as electron microscopy, histological techniques and specific detection of nucleic acid, immunofluorescence and immunoassays of both antigen and antibodies provide putative positive identification; an orthogonal approach using multiple assay types can be used to increase confidence in results.^8 30 31^ Individual IgM immune response can be also used as a diagnostic tool but is usually only performed during the convalescent phase of the illness; IgG is generally only used for epidemiological surveillance. Generally, the preferred method for diagnosing ebolaviruses and MARV is via direct detection of viral RNA using nucleic acid amplification testing (NAAT), with rapid diagnostic tests for EBOV antigen useful for cadaver testing.^32^

### Molecular diagnostics

Common targets for both ebolaviruses and MARV NAAT testing include the NP, L and GP genes.^33^ The gene sequence of GP is strain specific and therefore could be used for differentiation between infecting species; the sequences of VP40 and NP are more conserved. As recently published by Clark *et al*, there are a number of laboratory designed tests (LDTs) using reverse transcriptase (RT)-PCR, quantitative RT-PCR (qRT-PCR) and RT-loop mediated isothermal amplification to detect ebolaviruses and MARV,^34^ as diagnostic testing has historically been carried out in international reference laboratories with varying performance when evaluated by independent external quality control proficiency testing.^32 33 35^

The 2014–2016 West Africa ebolavirus outbreak resulted in the rapid development of new commercially available benchtop and point-of-care (POC, ie, fully automated platforms ideal for use in low-resource sites) NAATs to allow for rapid detection of ebolavirus infection in lower resource laboratories.^36–41^ Many of these tests are available for purchase and are approved by a stringent regulatory authority (SRA), either through WHO Emergency Use Authorization and listing (WHO EUAL), US Food and Drug Administration (FDA) Emergency Use Authorization (FDA EUA) or European Commission (CE) marking.^36^ At this time, there are only two MARV diagnostics approved by SRAs, mainly as part of a panel of assays with the ability to detect and differentiate between multiple pathogens.^36 41^

Quantification of the viral load is useful for patient management as it can potentially predict the probability of recovery since fatal cases often exhibit rapid increases in viraemia with viral load in blood for ebolaviruses reaching approximately 10^9^ copies/mL, while survivors’ viral titre ranges around 10^7^ copies/mL.^42^ Quantitative detection is achievable with several real-time RT-PCR-based methodologies (online supplementary tables S1 and S2).

A major limitation of the majority of the available NAATs is their limited strain coverage. Only a few NAATs are able to test for multiple ebolavirus or MARV subtypes, which can potentially result in false negatives during outbreak responses or surveillance activities. Further development is needed for easily deployable, POC NAATs that can distinguish multiple ebolavirus and MARV subtypes for future outbreaks and improved surveillance.

### Serological assays

**Figure 3:**
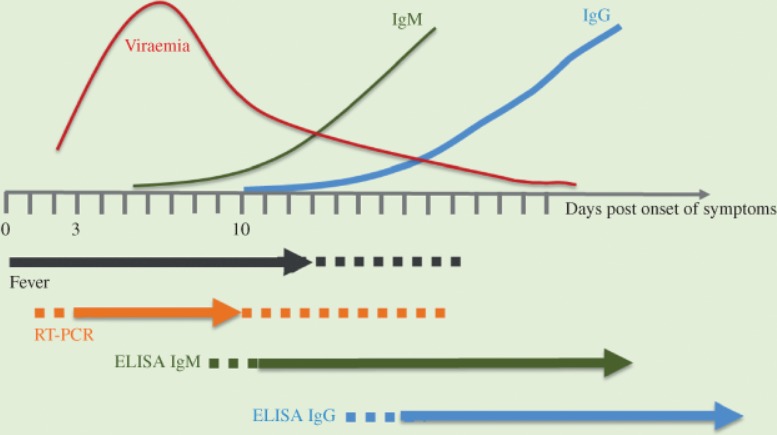
Diagnostic methodology and phase of illness of filovirus disease. *Reprinted from J Pathology, 235(2), Martines RB et al., Tissue and cellular tropism, pathology and pathogenesis of Ebola and Marburg viruses, 153–74. Copyright (2014), with permission from John Wiley and Sons.* ﻿RT-PCR, reverse transcriptase PCR.

Serology is useful in epidemiological studies of infected hosts by detection of ebolavirus-specific or MARV-specific antigen or IgM and IgG antibodies. Detection of anti-ebolavirus-IgM or anti-MARV-IgM indicates recent infection and can be detected as early as 2–4 days after symptom onset, while anti- ebolavirus-IgG or anti-MARV-IgG can be detected around 8–10 days after symptom onset and persist for up to 2 years after infection (figure 3). IgG detection is primarily used to identify individuals who have recovered from EVD or MVD or to assess seroprevalence in the community.^8 32 43–46^

There are currently no SRA-approved benchtop ELISAs for ebolavirus or MARV detection. Two commercial ELISAs are available for research use only to detect EBOV VP40 (online supplementary table S1) and IgG/IgM, and two LDTs are available to detect EBOV IgG/IgM.^31^ While ELISAs were developed for EBOV-specific antigen detection, RT-PCR is recommended for ebolavirus detection. For MARV, one ELISA LDT is available for viral detection.^47^

10.1136/bmjgh-2018-001112.supp1 Supplementary data

### Rapid diagnostics

Rapid diagnostic tests (RDTs, also called immunochromatographic tests or lateral flow immunoassays) can leverage the same antibody/antigen capture agents as an ELISA but in a lateral flow strip format, allowing faster results with minimal specimen processing (although proper infection control practices are still required, and more definitive identification may be necessary depending on the assay’s limit of detection and clinical sensitivity).^48^ RDTs are ideal as screening tests, suitable for field testing and low infrastructure settings such as the clinic.^32^ During the 2014–2016 ebolavirus outbreak, rapid screening of cadavers to support local burial practice was critical in controlling the outbreak and preventing the spread of virus.

The 2014–2016 ebolavirus outbreak resulted in the rapid development of new RDTs. As of May 2018, five EBOV RDTs were approved for the detection of EBOV by SRAs (either through WHO EUAL, FDA EUA or CE marking),^36 49 50^ with new tests in development.^51^ At this time, there are no MARV RDTs approved by SRAs.

Implementation requirements of the different filovirus diagnostic types are shown in table 1. Molecular diagnostics typically require the highest laboratory infrastructure, including biosafety hoods and a clean room, while most ELISAs can be run on the benchtop in more modest laboratory environments (providing suitable PPE is used when handling samples). POC or near-POC NAAT tests are generally automated and robust, and may be performed without a biosafety hood depending on sample preparation requirements. RDTs are typically designed for field or clinic use.

**Table 1:** EVD and MVD diagnostic comparisons

Test type	Laboratory infrastructure requirement (example)	Training requirement (example)	Turnaround time	In-house/prototype available for EVD/MVD	Commercial source available for EVD/MVD
Viral isolation, histology	HIGH (BSL-4 reference laboratory)	HIGH (advanced laboratory technician)	7–10 days	–	–
NAAT reference (including multiplex)	MODERATE/HIGH (reference laboratory)	MODERATE/HIGH (advanced laboratory technician)	3 hours (1–2 hours preparation)	Y/Y	Y/Y
NAAT POC	MODERATE (district hospital)	MODERATE (laboratory technician)	1–2 hours	Y/N	N/N
Serology (eg, ELISA)	MODERATE/HIGH (regional laboratory, district hospital)	MODERATE (laboratory technician)	3–4 hours	Y/Y	N/N
RDTs	LOW (clinic, health centre, field setting)	LOW (nurse, healthcare worker)	<30 min	Y/N	N/N

BSL-4, biosafety level 4; EVD, ebolavirus disease; MVD, Marburg virus disease; NAAT, nucleic acid amplification test; POC, point of care;RAD, rapid diagnostic test.

### Syndromic multiplex approach

Viral haemorrhagic fever (VHF) refers to a diverse group of animal and human illnesses in which fever and haemorrhage are caused by viral infection. In regions where VHF viruses may be endemic and maintained through natural reservoirs, a panel for distinguishing the viral family causing infection and, potentially, co-infection will be useful for surveillance as well as appropriate isolation and infection prevention and control measures. While the majority of ebolavirus outbreaks are caused by EBOV and SUDV, current tests do not have full strain coverage. A multiplexed, syndromic approach is a more useful and efficient strategy for surveillance, differential diagnosis and outbreak determination than relying on single, pathogen-specific assays. Currently, there are few commercially available molecular diagnostics that can detect multiple fever-causing agents.^41 52^

## Challenges in filovirus diagnostic development

### Lack of a regulatory approval pathway for filovirus diagnostics

During the 2014–2016 West Africa ebolavirus outbreak, WHO established an EUAL procedure for in vitro diagnostics (IVDs) in order to expedite the introduction of critically needed technologies during an outbreak setting.^53^ Similarly, the US FDA developed an EUA programme to expedite the approval process of American-made products.^54^

However, on 29 March 2016, WHO announced that the ebolavirus situation in West Africa no longer constituted a Public Health Emergency of International Concern. While previously submitted and approved products under WHO EUAL may still be purchased and used, both WHO and FDA recommend test manufacturers to submit for full regulatory approval.^53 54^ However, the high cost, limited availability of well-characterised clinical samples and time needed for resubmission may deter manufacturers from pursuing additional regulatory approvals of their existing product or new manufacturers to submit their product. This challenge, coupled with the evolving CE-IVD marking system in Europe,^55^ makes the diagnostic regulatory pathway for filoviruses and other emerging threats unclear.

In addition, as the ebolavirus outbreak waned, so has access to confirmed positive samples, which are required in order to fully validate an assay for regulatory approval. These circumstances make the regulatory approval process after an outbreak a significant challenge that must be addressed in order to ensure access to reliable and robust diagnostics in the future.

### Need for improved sensitivity and specificity of screening and triage tools

The need for rapid, sensitive, safe and simple ebolavirus diagnostic tests was highlighted in a November 2014 Call for Diagnostics by WHO since efforts to contain the ebolavirus outbreak were hampered by cumbersome, slow, complex and costly diagnostic tests. In order to stimulate product development, WHO issued a detailed product profile of the ‘ideal’ rapid, sensitive, safe and simple diagnostic test considered most likely to accelerate interruption of virus transmission in severely resource-constrained settings.^56^ It was suggested that the ‘ideal’ test should be designed for use in peripheral health clinics with no laboratory infrastructure in place, no biosafety precautions beyond the wearing of PPE, with three or fewer steps, and a time-to-result less than 30 min. Shortly after the WHO Call for Diagnostics was published, a target product profile (TPP) for ebolavirus diagnostics was developed by FIND, MSF, WHO and partners in 2014.^57^

Of the commercially available tests with publicly available performance data, two ebolavirus tests listed meet the ‘acceptable’ TPP criteria (ie, sensitivity >95% and specificity >99%) and one meets the ‘desired’ TPP criteria (ie, sensitivity >98% and specificity >99%).^40 41^ When surveillance testing in centralised settings is considered, without the decentralisation criteria, 10 tests meet the ‘acceptable’ criteria as listed in the TPP, including two LDTs. No POC tests meet the ‘desired’ or ‘acceptable’ criteria listed in the TPP. The RDT did not meet the sensitivity or specificity criteria when used with whole blood and just missed the cut-off for sensitivity when used to test buccal swabs (94%).^48^ The other POC tests did not meet the sensitivity criteria. If rapid, simple tests are needed most during an outbreak, one near-POC test is available, but POC tests that meet the TPP performance criteria are still needed. A number of laboratory-designed RDTs are under development that may meet these TPPs criteria (online supplementary table S1).

In terms of MARV, although a TPP for diagnostic tests to detect MARV does not exist, it may be helpful to extend the ebolavirus TPP criteria to evaluate the MARV tests, considering the viruses are within the same family and have many overlapping characteristics. Unfortunately, none of the MARV tests would be classified as ‘acceptable’ according to the ebolavirus TPP (although performance data are limited) due to the lack of deployable tests in areas with limited infrastructure and resources.

### Need for refined testing algorithms

Specifically for MARV, there are limited recommendations from WHO regarding the diagnosis of MVD. The Centers for Disease Control and Prevention recommends that ELISA, PCR or virus isolation be used to diagnose MVD within a few days of symptom onset. The IgG-capture ELISA is recommended when testing patients later in the course of the disease or post-recovery.^58^ Further refinement of MARV testing algorithms, especially as more sensitive diagnostics tests become available, should be considered.

### Use of alternative sample types for diagnosis

The usual clinical samples for testing for EVD and MVD are WB, plasma and serum. However, venepuncture is an invasive procedure with high risk for both the patient and the health worker. Moreover, blood separation into plasma or serum often requires additional equipment (eg, centrifuges) or expensive collection tubes. With the difficulties of blood collection and POC analysis under field conditions, less invasive, simpler to obtain and more stable clinical specimens must be considered for new diagnostic development.

There is evidence that ebolaviruses can also be detected in saliva, breast milk and semen.^59–62^ In the case of an outbreak in hard-to-reach areas, buccal swab testing to confirm fatality due to filovirus infection is important to inform behaviour in the field (eg, burial method, contact tracing). However, there is only one POC test that can accept oral fluids.^38^ Increasing the performance of RDTs to be used for screening via oral fluid from living individuals could provide a rapid triage test that could ultimately decrease the risk of infection for healthcare workers and others within the community, clinic and/or healthcare facility.

## Limited funding mechanisms and leveraging existing supply chain

In an effort to avoid manufacturer fatigue and promote development of novel diagnostics for outbreak response, new funding mechanisms and partnership models should also be considered. Many developers who commenced development of a diagnostic test for the detection of EVD ceased development as the 2014–2016 ebolavirus outbreak waned, as access to samples became challenging and funding slowed. Incentives such as advanced market commitments or volume guarantees for manufacturing scale-up could be considered in order to stimulate innovation in disease areas with small and volatile markets, such as EVD and MVD,^63^ and make it cost-effective for manufacturers who have invested in product lines for these disease areas to switch over their manufacturing lines to produce small batches of product when the need arises.

Leveraging existing supply chains, operator training on a specific platform technology, and product registration and regulatory experience of manufacturers with multiple tests on the market may also optimise programme efficiency for ebolavirus and MARV disease surveillance and outbreak management. In particular, platform technologies commercialised by manufacturers with products on the market for common infections and chronic diseases that require routine purchasing and training (ie, HIV) could be highly cost-effective and improve outbreak response times.

## Conclusion

The magnitude of the 2014–2016 West Africa ebolavirus outbreak brought to light the need for new diagnostic tests to rapidly assess and respond to VHF outbreaks to interrupt transmission. This review has identified several commercially available diagnostics and laboratory designed tests for filovirus detection.

However, significant gaps still remain (online supplementary figure S1 and figure S2). While there are EVD diagnostic tests approved or previously approved by SRAs and manufacturing scalability available for the detection of EBOV, additional test solutions for MVD and non-Zaire species of ebolavirus could better enable detection and response efforts in the future. Early triage and treatment of patients with EVD and MVD would have a number of benefits, including reduced nosocomial transmission and increased availability of hospital beds, leading to higher likelihood of survival and substantial reductions in the scale of outbreaks; however, because of the non-specific symptoms at early onset of disease, the need to rule out other aetiological agents for fever early during infection is important. RDTs with improved sensitivity and specificity would therefore be beneficial, and development of multiplexed diagnostic tests, especially those for use at POC, would better equip us to rapidly respond to future outbreaks. Another impactful way to affect the outcomes of future outbreaks would be to decrease opportunities for new infections among healthcare workers and sample/patient transporters by bringing tests and/or sample inactivation methods closer to the bedside.

10.1136/bmjgh-2018-001112.supp3 Supplementary data

10.1136/bmjgh-2018-001112.supp4 Supplementary data

In regions of the world where infrastructure and resources are limited or in situations where rapid response to decrease transmission is a priority, the use of simple, deployable tests is needed. Concerted, collaborative efforts in research, development and systems strengthening must occur to rapidly halt transmission and to prevent future transmission of filovirus diseases.

10.1136/bmjgh-2018-001112.supp2 Supplementary data
