# Supplementary material for: Diagnostics for filovirus detection: impact of recent outbreaks on the diagnostic landscape
Source: BMJ Glob Health. 2019 Feb 7;4(Suppl 2):e001112. doi: 10.1136/bmjgh-2018-001112 (PMC6407532; doi:10.1136/bmjgh-2018-001112)
Supplement: Supplementary data [file bmjgh-2018-001112supp001.pdf]

Table S1: Summary of Technology Assessments for EBOV

Commercial and regulated assays for EBOV are presented, as well as laboratory designed tests. Legacy in-house assays without formal regulation or clinical validation are not presented here, as they have limited or dated information for sensitivity/specificity/LOD.

Developer	System	Regulatory status	Sample type	Target	LOD	Sensitivity/PPA	Specificity/NPA	Specimens tested	Reference Assay	Capability to multiplex
**Molecular Diagnostics**										
**Alere (now Abbott)**	Alere q Filovirus Detect (w/ Alere q platform)	N/A[1]	Venous or finger stick blood,	RNA, L gene	no info	no info	no info	no info	no info	yes
**Altona Diagnostics GmbH (Ger)**	RealStar® Filovirus Screen RT-PCR Kit 1.0 (Human pathogenic Filovirus species)	WHO EUAL, FDA EUA	Plasma	RNA	1250 copies/mL	82%	100%	328 plasmas	Trombley	yes
**AmpliSense/Ecoli**	AmpiSensEBOV Zaire-FRT	RUO	All	RNA	2.00×10^3 GM/mL	95%	100%	no info	no info	no info
**Biocartis**	Idylla Ebola Virus Triage Test with Idylla Platform	FDA EUA, N/A[2]	Whole blood	RNA	216 pfu/mL or 178 copies/mL	97%	100%	Spiked samples	DoD Ebola Zaire (EZ1) rRT-PCR method on the ABI 7500 Fast Dx Real-Time PCR System	-
**Biomerieux**	FilmArray BioThreat E (ZEBOV only), BioThreat Kit (16 pathogen multiplex) Global Fever Panel	WHO EUAL (ZEBOV), RUO	Venous blood, urine	RNA	6.00×10^5 pfu/mL (WB), 6.00×10^3 for spiked samples	96%	100%	no info	Whole blood (WB), spiked samples	yes

[1] Product development ceased.
[2] Product purchasing unclear; development ceased.

Developer	System	Regulatory status	Sample type	Target	LOD	Sensitivity/PPA	Specificity/NPA	Specimens tested	Reference Assay	Capability to multiplex
**BioGene, Ltd. (UK)**	EbolaCheck on QuRapID platform	In dev	Whole blood	RNA, GP and human ribonuclease	1400 PFU/mL	no info	no info	Whole blood	Trombley	no info
**Bioneer (KOR)**	1. AccuPower® EBOV Real-Time RT-PCR Kit<br>2. AccuPower® EBOV Quantitative RT-PCR Kit	CE	Serum, plasma	RNA, NP Gene	47.86 copies/test	no info	no info	no info	no info	no
**Cepheid**	Xpert Ebola w/ XpertPlatform	WHO EUAL	Venous blood	RNA, NP and GP	0.13 pfu/mL	WB:100%, BS: 100%	WB: 99.5%, BS: 100%	whole blood, n=218; buccal swab, n=71	Trombley	yes
**Coyote Bioscience (CN)**	EBOV POC test with Mini-8 Real Time PCR platform	RUO	Whole blood	RNA	500 copies/mL	99.30%	97.90%	428 WB samples and 132 swab samples from corpses	no info	yes
**Genekam**	5 species-related Ebola RT-PCR kits	RUO	no info	RNA	no info	no info	no info	no info	no info	yes
**genesig (UK) (Primerdesign Ltd)**	Ebola 2014 Easy EBOV SUDV (genesig q16 PCR instrument)	RUO	All	RNA, NP	<100 copies/ mL	no info	100% (manufacturer claim)	no info	no info	yes
**Liferiver (Shanghai ZJ Bio-Tech Co.)**	Ebola Virus Real Time RT-PCR Kit	CE, WHO EUAL	Plasma, serum, whole blood, oral swab	RNA, EBOV + Zaire specific	23.9 copies/reaction	-	no info	no info	In-house	yes

Developer	System	Regulatory status	Sample type	Target	LOD	Sensitivity/PPA	Specificity/NPA	Specimens tested	Reference Assay	Capability to multiplex
**Lipsdiag**	LipsGene SUDV	RUO	Serum, plasma, amniotic or synovial fluid	RNA	no info	no info	100% (manufacturer claim)	no info	no info	yes
**Lucigen**	N/A	in dev	Fingerstick blood, cheek?	RNA, NP	no info	no info	no info	no info	in-house	no info
**MicoNanobiosys**	Ebola RT-PCR kit for Nanobiosys Sample Prep G2-16TU, Nanobiosys Real-time PCR G2-4	in dev	no info	RNA	no info	no info	no info	no info	no info	no info
**Optigene/Toshiba Medical Systems**	EBOV by RT-LAMP	RUO	no info	RNA	no info	100% and 97.9% for the trailer and nucleoprotein primer sets, respectively	100%	100 (serum, n=44; oral swab, n=56)	no info	no info
**Sacace Biotechnologies**	Ebola Zaire Real-TM (for use w/ SaMag Automatic Nucleic Acid Extraction instrument	RUO	Whole Blood, Plasma , Saliva, Tissue	NP protein	>400 copies/mL	no info	100%	no info	no info	yes
**ThermoFisher**	Ebola Virus NP Real-Time PCR Assay (CDC Assay)	FDA EUA, RUO	Whole blood, serum, plasma	RNA, NP	62.5 copies/mL	99.80%	no info	no info	Altona + 9 other assays	no info
**TIB MolBio (USA)**	LightMix Ebola Zaire rRT-PCR Test (TIB Molbiol) w/ LightCycler (Roche)	FDA EUA, RUO	Whole blood	RNA	4,781 pfu/mL	95%	100%	no info	no info	no
**GenArraytion Inc. for Luminex xMAP or TaqMan**	BioThreat MULTIFLEX™, Febrile Associated Pathogens MULTIFLEX® 2	RUO	All	RNA	no info	no info	no info	no info	no info	yes

Developer	System	Regulatory status	Sample type	Target	LOD	Sensitivity/PPA	Specificity/NPA	Specimens tested	Reference Assay	Capability to multiplex
**Serology**										
**Zalgen Labs, LLC**	ReEBOV VP40 Antigen ELISA test kit	RUO	Whole blood, serum	VP40 protein	no info	no info	no info	no info	no info	no info
**Zalgen Labs, LLC**	ReEBOV IgG/IgM ELISA test kit	RUO	Whole blood, serum	no info	no info	no info	no info	no info	no info	no info
**RDTs**										
**BIOCREDIT/Rapigen**	Biocredit Ebola Ag Rapigen	RUO	Whole blood, plasma, or serum	no info	no info	no info	no info	no info	no info	no info
**InTec (CN)**	One Step Ebola Test	RUO	Whole blood, plasma, or serum	no info	no info	ALTONA: Plasma: 98.43% TROMBLEY: plasma: 86.61%	ALTONA: Plasma: 80.2% and whole blood: 95% TROMBLEY: plasma - 84.71%	-	Altona + Trombley	yes
**Lifeassay**	N/A	RUO	no info	no info	no info	no info	no info	no info	no info	no info
**Orasure (USA)**	OraQuick Ebola Rapid Antigen Test	WHO EUAL, FDA EUA	Oral fluid, whole blood	VP40 antigen	53,000 pg/mL	84% (whole blood) 94% (buccal swab)	98% (whole blood), 100%(buccal swab)	244 buccal swab samples, 75 whole blood samples	-	-
**Otsuka Pharmaceutical Co., Ltd. (JPN)**	QuickNavi-EBOLA	In dev	no info	no info	no info	no info	no info	no info	no info	no info
**SD Biosensor (KOR)**	SD Q Line Ebola Zaire Ag	WHO EUAL	Plasma, serum, fingerstick	GP, NP, VP40	no info	ALTONA: Plasma: 84.5% TROMBLEY: plasma:70.511%	ALTONA: Plasma: 98.99% and whole blood:100% TROMBLEY: plasma - 99.42%	1) 100 whole blood and 346 stored plasmas	1) Trombley + Altona	yes

Developer	System	Regulatory status	Sample type	Target	LOD	Sensitivity/PPA	Specificity/NPA	Specimens tested	Reference Assay	Capability to multiplex
**Senova**	DEDIATEST-Ebola	CE	Serum, plasma, swab?	VP40 Ag	106 pfu/mL	79.53% on plasmas (compared to altona) and 70.13% on plasmas (compared to Trombley)	100% on whole blood, 84.24 % on plasmas (compared to Altona) and 85.96% on plasmas, (compared to Trombley)	1) 100 fresh whole blood samples, 346 stored plasma samples; 2) 115 plasmas	1) Altona + Trombley. 2) Luft antibodies - JID 2007; 196 (S2): S184-S192	
**Vedalab/CEA**	eZYSCREEN®	CE	Blood, plasma, or urine	no info	no info	no info	no info	no info	no info	no
**Zalgen Labs, LLC/Coregenix**	ReEBOV Antigen Rapid Test	WHO EUAL	Fingerstick, venous, serum, plasma	VP40 antigen	$2.11\text{x}10^8$ copies/mL	1) PLASMA Altona: 93.18% Trombley: 85.26%; 2) 91.8%; 3) 91%	1) WHOLE BLOOD Altona: 98% PLASMA Altona: 80.3% Trombley: 83.04%; 2) 84.6%; 3) stored plasma: 93%	1) 100 fresh whole blood samples and 346 stored plasma samples; 2) 147 fresh whole blood, 146 frozen plasma; 3) 83 stored plasma	1) Altona + Trombley; 2) unknown; 3) CDC in-house assay	no
**LDTs**										
**CDC Assay**	Ebola Virus VP Real-Time PCR Assay	N/A	RNA	VP protein	600 TCID50/mL	no info	100.00%	no info	no info	N/A
**DOD Assay -- DTRA CB/JSTO**	DoD Ebola Zaire (EZ1) rRT-PCR (TaqMan®) Assay	N/A	-	RNA	1000 pfu/mL	no info	no info	Spiked samples	no info	N/A
**Integrated DNA Technologies (IDT)**	IDT PrimeTime qPCR Assay	N/A	-	RNA	36 copies/mL	98.90%	no info	no info	no info	yes
**Trombley -- Porton Down assay**	Trombley	N/A	serum and plasma	RNA	no info	100%	100%	328 plasmas	altona filovirus assay	N/A
**CDC ELISA**	IgM ELISA<br>IgG ELISA	N/A	Serum	IgM, IgG	no info	no info	no info	no info	no info	no info

Developer	System	Regulatory status	Sample type	Target	LOD	Sensitivity/PPA	Specificity/NPA	Specimens tested	Reference Assay	Capability to multiplex
**Public Health Canada**	IgG ELISA	N/A	Serum	IgG	no info	no info	no info	no info	no info	no info
**Defence Science and Technology Laboratory (DSTL)**	EBOV lateral flow assay	N/A	Capillary blood	no info	no info	no info	no info	131 fresh whole blood samples (capillary and venous blood)	Altona RealStar® Filovirus RT-PCR	N/A
**Naval Medical Research Center (NMRC)**	EBOV lateral flow immunoassay	N/A	Blood samples, oral swabs	no info	no info	no info	no info	290 plasma samples, 237 oral swabs	rRT-PCR GP and NP assays	N/A
