# Supplementary material for: Diagnostics for filovirus detection: impact of recent outbreaks on the diagnostic landscape
Source: BMJ Glob Health. 2019 Feb 7;4(Suppl 2):e001112. doi: 10.1136/bmjgh-2018-001112 (PMC6407532; doi:10.1136/bmjgh-2018-001112)
Supplement: Supplementary data [file bmjgh-2018-001112supp003.pdf]

Figure S1: Ebolavirus Heat Map - Diagnostics available or needed for the key use cases

Ebola	*Target Use Setting*						
	**Facility-based testing and recommended diagnostic technology**						**Non-facility-based testing**
Intended Use of Test (Use Case)	4. National Reference Lab	3. Regional/ Provincial Lab	2. District Hospitals			1. Health Centers	0. Health Posts, Field Settings
	NAAT, PRNT, ELISA	NAAT, ELISA	POC NAAT	ELISA	RDT	RDT	RDT
**Non-outbreak setting**							
**Surveillance** Diagnostics support syndromic surveillance systems (by adding specificity through diagnostic testing)	NAAT [1] ELISA, sequencing	NAAT [1] ELISA	Not cost effective	IgG, IgM, Ag ELISA ǂ	ELISA more sensitive	Multiple Ag RDTs [2]	Multiple Ag RDTs [2]
**Syndromic diagnosis** Clinical setting - detection of pathogen in syndromic case management (multiplex/panel diagnostics)	PCR panels available	PCR panels available	In development [1]	IgM, Ag LDTs	Multiple Ag RDTs [2]	Multiple Ag RDTs [2]	Multiple Ag RDTs [2]
**Case management** Confirmation of pathogen	NAAT [1] sequencing, virus isolation	NAAT [1] IgM, Ag ELISA	In development [1]	IgG, IgM, Ag ELISA ǂ	RDT screening only	RDT screening only	RDT screening only
**Outbreak setting**							
**Case management** Diagnosis/confirmation of pathogen	NAAT [1,2] sequencing, virus isolation	NAAT [1,2] IgM, Ag ELISA [2]	In development [1]	IgG, IgM, Ag ELISA ǂ	Multiple Ag RDTs [2]	Multiple Ag RDTs [2]	Multiple Ag RDTs [2]
**Diagnostics stage legend:**							
Platforms/tests available							
Lab/field trials ongoing or completed							
Under development							
Needed/needs improvement							
Not needed/not useful							

[1] Some NAAT/PCR tests may be region/strain specific; may require multiple tests or sequencing to ensure detection sensitivity

[2] Requires regulatory approval (may only have emergency authorization for detection/diagnosis)

ǂ IgG more often used for detection in convalescent or post-infection phase
