# Supplementary material for: Diagnostics for filovirus detection: impact of recent outbreaks on the diagnostic landscape
Source: BMJ Glob Health. 2019 Feb 7;4(Suppl 2):e001112. doi: 10.1136/bmjgh-2018-001112 (PMC6407532; doi:10.1136/bmjgh-2018-001112)
Supplement: Supplementary data [file bmjgh-2018-001112supp002.pdf]

Table S2: Summary of Technology Assessments for MARV

Commercial and regulated assays for MARV are presented, as well as laboratory designed tests. Legacy in-house assays without formal regulation or clinical validation are not presented here, as they have limited or dated information for sensitivity/specificity/LOD.

Developer	System	Regulatory status	Sample type	Target	LOD	Sensitivity/PPA	Specificity/NPA	Specimens tested	Reference Assay	Capability to multiplex
**Molecular Diagnostics**										
**Alere (now Abbott)**	Alere q Filovirus Detect (w/ Alere q platform)	in dev	Venous or fingerstick blood	RNA	no info	no info	no info	no info	no info	yes
**Altona Diagnostics GmbH (Ger)**	RealStar Filovirus RT-PCR kit	WHO EUAL	Plasma	RNA	1.1 copies/uL for MARV Popp and 4.2 copies/uL for MARV Musoke	no info	no info	no info	no info	yes
**Biomerieux**	Film Array BioThreat kit (16 pathogen multiplex)	RUO	Venous, urine	RNA, 2 Targets	no info	no info	no info	no info	no info	yes
**Genekam**	Ebola/Marburg test	RUO	-	RNA	no info	no info	no info	no info	no info	yes
**Liferiver (Shanghai ZJ Bio-Tech Co.)**	Marburg Virus (MBV) Real Time RT-PCR Kit	CE	Plasma, serum, whole blood	RNA	no info	no info	no info	no info	no info	yes
**GenArraytion Inc. for Luminex xMAP or TaqMan**	BioThreat MULTIFLEX™, Febrile Associated Pathogens MULTIFLEX® 2	RUO	RNA	RNA	no info	no info	no info	no info	no info	yes
**LDTs**										
**Center for Disease Control**	TaqMan Array Card (TAC)	N/A	Whole blood	no info	no info	88%	99%	1050	no info	N/A
**NRI Japan and PHAC**	Bench-top RT-LAMP	N/A	no info	no info	100 copies/tube	78%	100%	24	no info	N/A
**Public Health Agency Canada**	Benchtop ELISA	N/A	no info	no info	~10 pfu/well	no info	no info	no info	no info	yes
